# Evaluation of the Growth, Sporulation, Fungicide Efficacy, and Host Range of *Ramularia sphaeroidea*

**DOI:** 10.3390/microorganisms12040766

**Published:** 2024-04-10

**Authors:** Min Shi, Yan-Zhong Li

**Affiliations:** 1 State Key Laboratory of Herbage Improvement and Grassland Agro-Ecosystems, College of Pastoral Agriculture Science and Technology, Lanzhou University, Lanzhou 730000, China; shim19@lzu.edu.cn; 2 Engineering Research Center of Grassland Industry, Ministry of Education, Lanzhou 730020, China

**Keywords:** *Ramularia sphaeroidea*, hairy vetch, growth, sporulation, fungicide efficacy, host range

## Abstract

*Ramularia sphaeroidea* was primarily identified based on the characteristics of its conidia and several sequences. The fungus causes severe leaf spot disease on hairy vetch (*Vicia villosa* var. *glabrescens*) in Yunnan Province in China. The growth, sporulation, fungicide efficacy, and host range of the pathogen were evaluated to aid in disease management. Different types of culture media and carbon and nitrogen sources were used to evaluate the growth of *R. sphaeroidea.* Oatmeal, maltose, and potassium nitrate agar had a higher amount of sporulation. Difenoconazole (10%) was the most effective fungicide against the leaf disease caused by *R. sphaeroidea*. In addition, foliar inoculation sprays were used to assess the host range of *R. sphaeroidea* in six different plant species, including alfalfa (*Medicago sativa* L.), sainfoin (*Onobrychis viciifolia* Scop.), erect milkvetch (*Astragalus adsurgens* Pall.), common vetch (*Vicia sativa* L.), red clover (*Trifolium pratense* L.), and white clover (*Trifolium repens* L.). *R. sphaeroidea* successfully infected these plants, indicating that it has a wider host range than hairy vetches.

## 1. Introduction

The legume hairy vetch (*Vicia villosa* Roth var. *glabrescens*) is a variant of *Vicia villosa* Roth [1,2,3] and is also known as *Vicia villosa* Roth var. *glabrescens* [4]. It is native to Europe and western Asia, and it is also the second most common vetch cultivated in the world [5]. It is primarily found in the USA, Canada, Japan, Argentina, South Korea, and Iran [6,7,8]. It was introduced to China from the state of Oregon, USA, in 1946 and has been planted in Jiangsu, Anhui, Shandong, Henan, Hubei, Yunnan, and other provinces [3,9]. In addition, it was planted as a winter cover crop in Japan and South Korea. Leguminous cover crops are preferably cultivated, owing to their ability to harbor bacteria that fix nitrogen in nodules [7]. Compared with other cover crops, planting hairy vetch leads to improved soil quality and health [10,11].

Previous studies around the world have described more than 10 diseases in species of *Vicia*, including chocolate spot (*Botrytis fabae*) that infected the leaves of *V. fabae* [12], root rot caused by *Macrophomina phaseolina* and *Rhizoctonia solani* [13], and *Ramularia sphaeroidea* Sacc. in *V. villosa* Roth [14]. In China, Nan and Li [15] described 10 diseases of *V. villosa* Roth in 1994, including *Ascochyta pisi*, *Colletotrichum viciae*, *Leveillula leguminosarum* Golov., *Fusarium oxysporum* Schlecht., and *Stemphylium vesicarium*.

*Ramularia* is a rich genus that includes important plant pathogens such as *R. collo-cygni* and *R. beticola*, which caused severe economic losses to barley and sugar beet crops, respectively [16]. *Ramularia* leaf spot is an important disease on wheat, turfgrass, and perennial grasses and forms a potential threat to other agriculturally important crops [17]. Leaf spot disease caused by *R. collo-cygni* on barley has occurred in European countries and New Zealand, which can lead to yield losses of up to 25% in winter barley [18,19]. *R. collo-cygni* can also lead to symptomless infection through a special stomatopodium structure and epiphytic hyphal network during the entire barley growing season when it completes its life cycle [17,20].

*R. sphaeroidea* is also an important pathogen in *Ramularia,* which was first described by Braun in 1995, based on its hyaline conidiophores and conidia [21,22]. With the advent of molecular techniques, *R. sphaeroidea* was first identified in the second year [14]. In 2021, leaf spot disease caused by *R. sphaeroidea* in hairy vetch was reported for the first time in China [23].

The availability of different nutrients has a significant influence on the progression of diseases caused by pathogens [24,25]. Fungal growth and sporulation need a lot of elements, such as carbon, nitrogen, oxygen, and hydrogen [26]. Many nutrients are required, including carbohydrates, nucleic acids, and proteins, which are involved in host–pathogen interactions [27,28,29,30]. *R. sphaeroidea* has difficulties in inducing conidial production, which limits further study on its biological characteristics and effective control [14,23]. Therefore, it is essential to determine the optimum nutrient conditions for the growth and sporulation of *R. sphaeroidea*.

In 2002, Strobilurin-based fungicides were found to be the most effective fungicide to control the disease caused by *R. collo-cygni* [31]. Less than ten years later, there was a rapid decline in strobilurin-based fungicides for *Ramularia* disease because of resistance [32]. Azoxystrobin in combination with chlorothalonil was considered to be effective [33]. The high adaptive potential of *Ramularia* species led to the low efficacy of the current fungicides. To our knowledge, comprehensive identification and effective fungicides are lacking, and the effects of different nutrients on *R. sphaeroidea* are poorly understood. Understanding the host range and fungicide efficacy of the *R. sphaeroidea* strain from Yunnan could guide strategies to prevent the widespread dissemination of this disease in China. Therefore, this study was conducted to investigate the host range through artificial inoculation under greenhouse conditions, and the most effective fungicide against leaf diseases caused by *R. sphaeroidea* was identified. Furthermore, the effects of carbon and nitrogen on the colony growth and sporulation of *R. sphaeroidea* were evaluated. In addition, we conducted a more accurate phylogenetic analysis of the multigene sequences.

## 2. Materials and Methods

### 2.1. Isolation of the Fungus

Twenty diseased leaves (four leaves per plant) of *Vicia villosa* Roth var. *glabrescens* were randomly collected from Malong County (103°36′10″ E, 25°31′78″ N at an elevation of 1985.8 m) in 2019 [34]. The leaves were cut into pieces, soaked in 75% ethanol for 45 s and 1% sodium hypochlorite (NaClO) for 75 s, washed four times with sterile water, dried with sterilized filter paper, and then placed on potato dextrose agar (PDA) using sterilized forceps [35]. A total of 107 isolates were identified, and the frequency was 33.64%. Three representative isolates were selected for further analysis. The diseased specimen with the number MHLZU19326 and representative isolates (YN1931401, YN1931402, and YN1931403) were deposited at the Mycological Herbarium of Lanzhou University (MHLZU). Morphological characterizations of conidiophore and conidia were observed using a light microscope and photographed with a Canon DS126391 camera (Canon, Lanzhou, China).

### 2.2. Multigene Sequencing

A Fungal DNA Kit (D3195) purchased from Omega-BioTek (Norcross, GA, USA) was used to extract DNA from three isolates of *R. sphaeroidea.* The 28S rRNA gene (LSU), internal transcribed spacer regions (ITS), calmodulin (*cmdA*), translation elongation factor 1-α (*tef1-α*), histone H3 (*his3*), glyceraldehyde-3-phosphate dehydrogenase (*gapdh*), and RNA polymerase II second largest subunit (*rpb2*) genes were amplified and sequenced with the primers LR5/LSU1Fd [36,37], ITS4/V9G [38,39], CAL-228F/CAL-737R [40], EF1-728F/TEF-1R [41,42], CylH3F/CylH3R [43,44], GPD1/GPD2 [45], and RPB2-5f2/RPB2-7 [46,47], respectively. PCR was performed as previously described by Videira [16].

### 2.3. Growth, Sporulation, and Germination of R. sphaeroidea

#### 2.3.1. Culture Media

Five types of agars were used to test different media, including PDA, oatmeal agar (OMA), potato carrot agar (PCA), corn meal agar (CMA), and L-malic acid added to PDA [14,48]. Each medium was sterilized at 121 °C for 20 min and then cooled to room temperature. Each medium was poured into 90 mm diameter Petri dishes and solidified for 24 h in aseptic conditions.

#### 2.3.2. Carbon and Nitrogen Media

The components of the basal medium consisted of 2 g of sodium phosphate (Na_3_PO_4_), 1 g of magnesium sulfate (MgSO_4_), 5 g of potassium nitrate (KNO_3_), and 20 g of dextrose in different types of carbon and nitrogen media [48]. The six types of carbon media included D-fructose, glucose, sucrose, lactose, starch, and maltose, and the four types of nitrogen media included potassium nitrate, ammonium nitrate, peptone, and ammonium chloride. The pathogen was cultured on PDA for 45 days. Fungal plugs that were 5 mm in diameter were excised and placed in the middle of each Petri dish. All the plates were cultured at 25 °C.

Colony diameters were recorded after 7, 14, 21, 28, and 35 days of incubation. On day 35, the daily growth rate (mm day^−1^) was calculated, and a hemocytometer was used to calculate the concentration of conidial suspensions in each medium. The YN1931401 strain was used in this experiment, and four replicates were designed for each treatment.

#### 2.3.3. Effects of Temperature on Conidial Germination

Conidia were washed with distilled water from 45-day-old colonies growing on PDA, and the concentrations were adjusted to approximately 10^6^ conidia mL^−1^ with a hemocytometer. Three 10 μL droplets of conidial suspension were added to the PDA plate and incubated at temperatures ranging from 5 to 35 °C at 5 °C intervals. The Petri dishes were removed from the incubators at 24, 48, 72, and 96 h, respectively. The percentage germination of each treatment was evaluated by microscopy.

### 2.4. Phylogenetic Analysis

All reference sequences were downloaded from GenBank and listed in Supplementary Table S1 [49,50,51,52,53], as described by Videira et al. [16]. Each sequence was aligned using MEGA 7.0.2. The sequence was used to combine the seven loci using Matrix 1.8. MrModeltest v. 2.3 was used to select the best-fit nucleotide substitution models for phylogenetic analysis and added to MrBayes v. 3.2.6. The full dataset was then run for 2,000,000 generations and sampled every 100 generations. For maximum likelihood, the test of phylogeny was the bootstrap method, and the number of bootstrap replications was 1000.

### 2.5. Pathogenicity and Host Range

To test Koch’s postulates, a spray inoculation experiment was conducted in September 2021 to test the pathogenicity of *R. sphaeroidea*. The seeds of *Vicia villosa* var. *glabrescens* were obtained from the Academy of Grassland and Animal Science (Kunming, China) in 2018. *R. sphaeroidea* strain YN1931401, isolated from diseased hairy vetch plants, was used in this study. The pathogen was cultured at 25 °C for 45 days. About 10 mL conidial suspension (1 × 10^6^ conidia mL^−1^) containing Tween 80 (0.01%) was sprayed on the leaves of 25 plants, and an additional 25 plants were sprayed with sterile water as the control group.

Six locally grown perennial legumes, including sainfoin (*Onobrychis viciaefolia* Scop.), alfalfa (*Medicago sativa* L.), white clover (*Trifolium repens* L.), common vetch (*V. sativa* L.), erect milkvetch (*Astragalus adsurgens* Pall.), and red clover (*T. pratense* L.), were used to access the host range of the YN1931401 strain of *R. sphaeroidea* isolated from hairy vetch plants. In total, 120 plants were used in this experiment. Twenty seeds of each plant species were selected and surface-sterilized with 75% ethanol for 50 s and 1% sodium hypochlorite (NaClO) for 90 s, followed by rinsing three times with sterile water. Approximately 10 mL of a conidial suspension of *R. sphaeroidea* (1 × 10^6^ conidia mL^−1^) was sprayed on ten plants, and the same amount of sterile water was sprayed on the other ten control plants.

Polyethylene bags were used to cover all plants for 48 h to maintain humidity above 95%. After this, all the plants were placed randomly on a rack in a greenhouse under an 18 h/6 h (light/dark) regime with a temperature of 22 °C during the day and 18 °C at night. The incidence of infected leaves was evaluated 14 days after inoculation, and the pathogen was re-isolated from the infected lesions to confirm its identity using morphological approaches.

### 2.6. Fungicide Sensitivity Experiments

To determine the efficacy of four fungicides, including chlorothalonil (a.i. 75%, Sichuan, China), mancozeb (a.i. 80%, Shandong, China), difenoconazole (a.i. 10%, Zhejiang, China), and pentazole alcohol (a.i. 50%, Zhejiang, China), were used against *R. sphaeroidea*. Different concentrations of chlorothalonil (187.5, 375, 750, 1500, and 3000 mg/L), mancozeb (200, 400, 800, 1600, and 3200 mg/L), difenoconazole (200, 400, 800, 1600, and 3200 mg/L), and pentazole alcohol (200, 400, 800, 1600, and 3200 mg/L) were added to sterilized PDA media before solidifying. PDA without fungicide was used as a control. Fungal mycelium plugs with a 5 mm diameter were transferred to the center of a Petri dish containing various concentrations of fungicides. The colony diameter was measured using a ruler after two weeks [54] at a temperature of 22 °C. The mean was calculated based on four replicates, and regression analysis was conducted using percent inhibition values obtained from mycelium growth tests and logarithmic values of the fungicide doses. Subsequently, EC50 values were calculated [55].

## 3. Results

### 3.1. Phylogenetic Analysis

**Figure 1:**
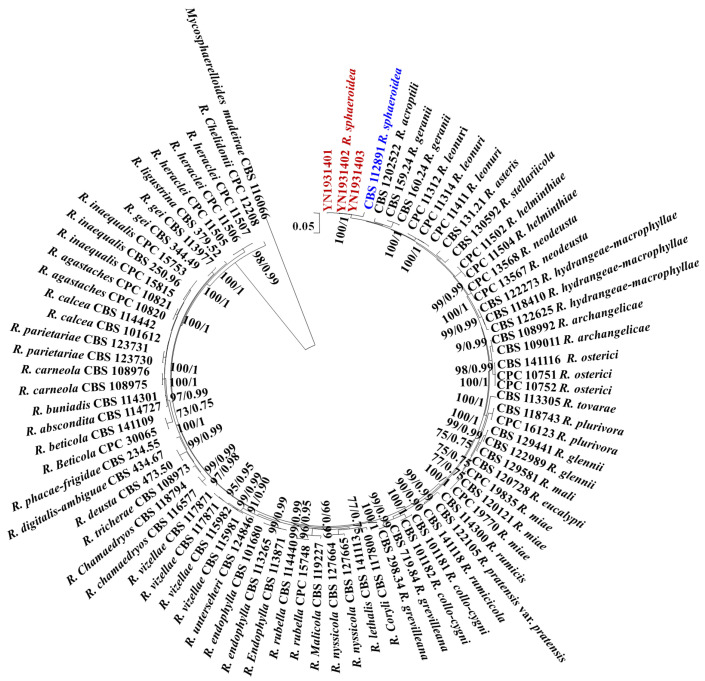
Maximum likelihood phylogenetic tree of concatenated partial sequences of ITS, LSU, *cmdA*, *gapdh*, *his3*, *rpb2,* and *tef1-α* gene alignments of *Ramularia* species. Bootstrap values for the maximum likelihood and Bayesian posterior probabilities are shown above the branches. *Mycosphaerelloides madeirae* (CBS 116066) is used as an outgroup. The isolates YN1931401, YN1931402, and YN1931403 in this study were marked in red and the *R. sphaeroidea* that reported in 2015 was marked in blue.

All sequences of the reference *Ramularia* species were downloaded from GenBank, and *Mycosphaerelloides madeirae* was used as the outgroup. The sequence contained 3155 characters (442 for ITS, 659 for LSU, 177 for *cmdA*, 578 for *gapdh*, 346 for *his3*, 574 for *rpb2*, and 379 for *tef1-α*). The following models were selected by MrModeltest 2.3 for MrBayes analysis: GTR+G for ITS, GTR+G for LSU, GTR+G for *cmdA*, GTR+I+G for *gapdh*, GTR+G for *his3*, GTR+G for *rpb2*, and SYM +G for *tef1-α*. The combined multigene tree constructed using ITS, LSU, *cmdA*, *gapdh*, *his3*, *rpb2,* and *tef1-α* indicated that the isolates YN1931401, YN1931402, and YN1931403 were *R. sphaeroidea* (Figure 1).

### 3.2. Symptoms, Pathogen Isolation, and Conidia Germination

**Figure 2:**
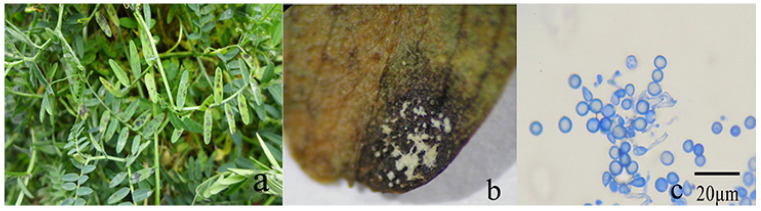
Leaf spot symptoms on hairy vetch plants: (**a**) field symptoms of leaf spot, (**b**) leaf symptoms of blackening and obvious white mold on the leaves, and (**c**) subglobose conidia directly found in leaves. Scale bar = 20 μm.

From 2019 to 2021, leaf spot disease in hairy vetch was observed between November and December in Malong County, Qujing City, Yunnan Province, China. The disease generally occurred in the older leaves. The initial symptoms were small, irregular, brown to dark brown spots (Figure 2a), which eventually developed into a white mass of mycelia in the middle portion of the lesions, with conidia that were found directly in the leaves (Figure 2b,c). The leaf lesions were initially dark and then fused with the surrounding black spots.

### 3.3. Growth, Sporulation, and Germination of R. sphaeroidea

#### 3.3.1. Culture Media

**Figure 3:**
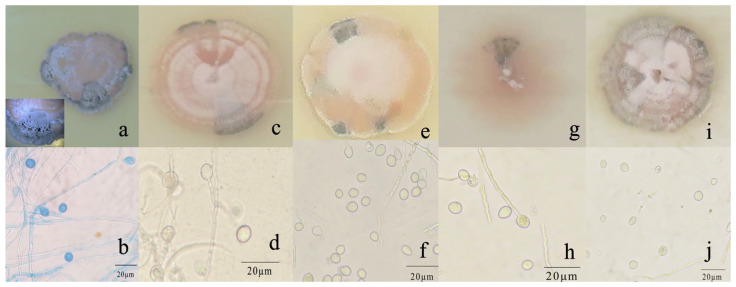
Morphological and cultural characteristics of *R*. *sphaeroidea*. (**a**,**c**,**e**,**g**,**i**) Colony morphology on PDA, OMA, LA-PDA, PCA, and CMA, respectively. (**b**,**d**,**f**,**h**,**j**) Subglobose to globose conidia on PDA, OMA, LA-PDA, PCA, and CMA, respectively. Scale bar = 20 μm.

**Figure 4:**
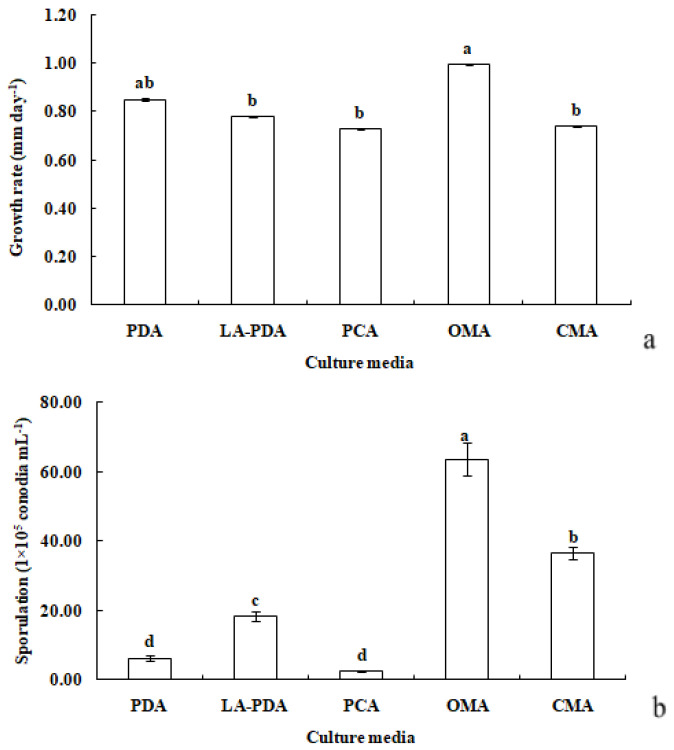
Growth rate (**a**) and sporulation (**b**) of *R*. *sphaeroidea* in five types of culture media. The same lowercase letter indicates no significant difference between treatments at *p* ≤ 0.05, according to Tukey’s HSD.

*R. sphaeroidea* grew and produced spores on all media tested (Figure 3). The conidia were aseptate on all media, and their shapes were ellipsoid to spherical (Table 1). Among the growth media tested, cultures grown on OMA grew the fastest at a rate of 1.0 mm per day followed by PDA with an average daily growth rate of 0.85 mm. Moreover, the colonies on both L-malic acid PDA and CMA grew an average of 0.78 and 0.74 day^−1^, respectively. The fungi cultured on PCA grew 0.73 mm day^−1^ on average, which was the lowest rate of growth compared with those on the other media (Figure 4a). The results from this study showed that OMA promoted the highest number of conidia with a suspension of 63.50 ×10^5^ conidia mL^−1^ followed by the numbers produced on CMA. Simultaneously, the lowest amount of sporulation was recorded on PCA followed by PDA (Figure 4b).

#### 3.3.2. Carbon Sources

**Figure 5:**
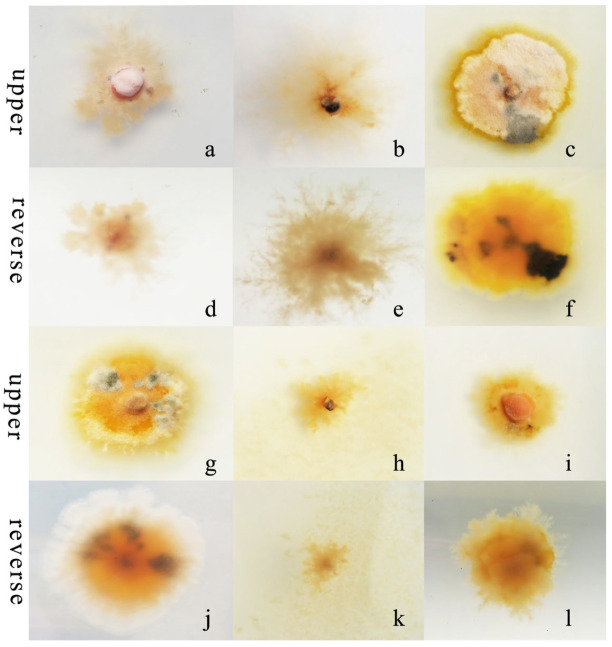
Morphological and cultural traits of *R. sphaeroidea*. (**a**–**c**,**g**–**i**) Colony morphology on the upper side of glucose, sucrose, lactose, D-fructose, starch, and maltose, respectively. (**d**–**f**,**j**–**l**) Colony morphology on the reverse side of glucose, sucrose, lactose, D-fructose, starch, and maltose, respectively.

**Figure 6:**
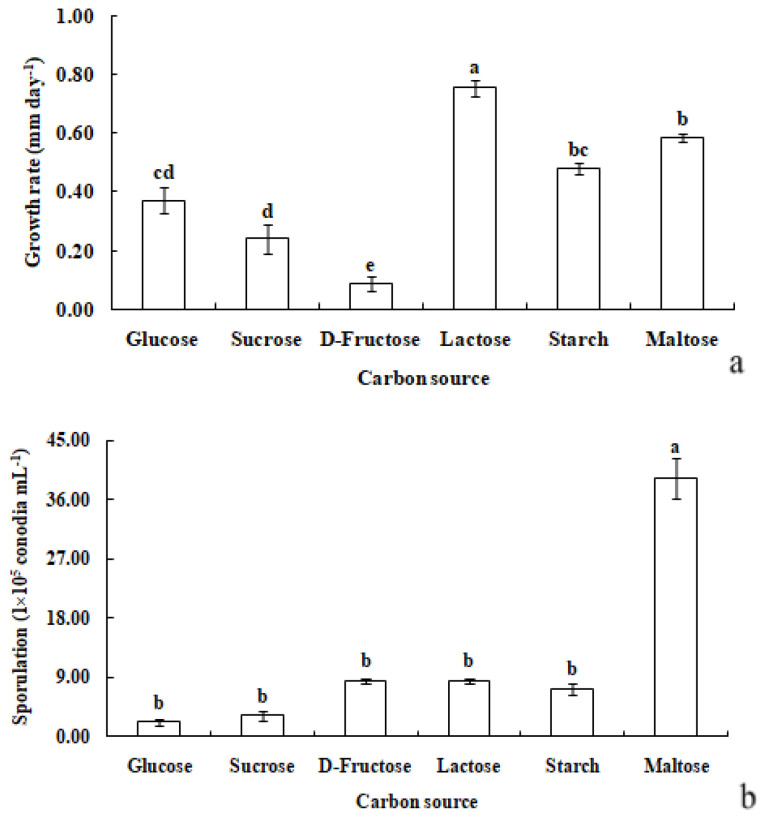
Growth rate (**a**) and sporulation (**b**) of *R. sphaeroidea* on six types of carbon media. The same lowercase letter indicates no significant difference between treatments at *p* ≤ 0.05 according to Tukey’s HSD.

*R. sphaeroidea* grew and produced spores in all the carbon media (Figure 5). It grew better on lactose and maltose with an average daily growth rate of 0.75 and 0.59 mm, respectively. The average growth rate on starch was 0.48 mm day^−1^ (Figure 6a). Moreover, the average growth on glucose was 0.37 mm day^−1^. It had the lowest growth rate on D-fructose of 0.09 mm day^−1^. No significant difference was observed in its growth on starch, maltose, glucose, and sucrose (Figure 6a). The pathogen sporulated prolifically on maltose, which was followed by lactose, D-fructose, and starch. However, the amount of sporulation was significantly reduced on glucose and sucrose (Figure 6b).

#### 3.3.3. Nitrogen Sources

**Figure 7:**
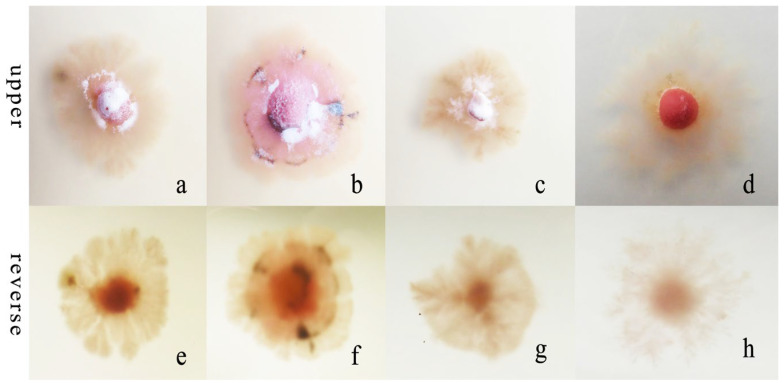
Morphological and cultural characteristics of *R. sphaeroidea*. (**a**–**d**) Colony morphology on the upper side of media that contained potassium nitrate, ammonium nitrate, ammonium chloride, and peptone, respectively. (**e**–**h**) Colony morphology on the reverse side.

**Figure 8:**
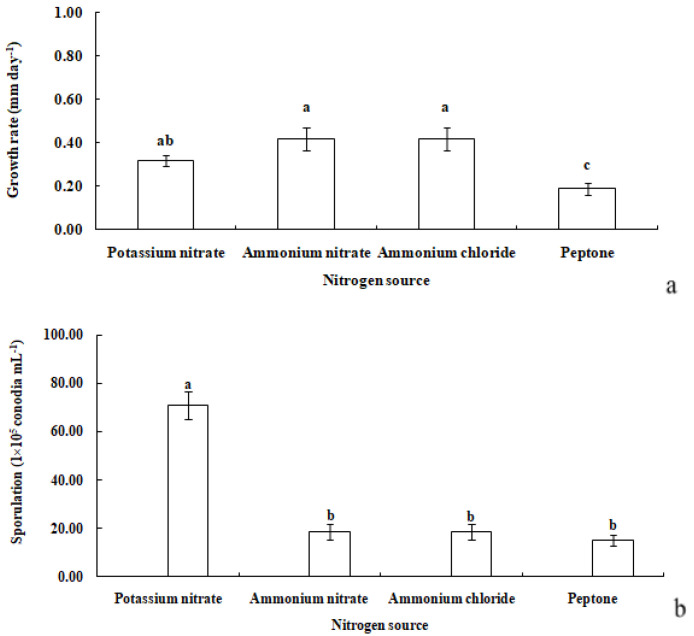
Growth rate (**a**) and sporulation (**b**) of *R. sphaeroidea* on four types of nitrogen media. The same lowercase letter indicates no significant difference between treatments at *p* ≤ 0.05 according to Tukey’s HSD.

*R. sphaeroidea* grew and produced spores on four types of nitrogen media (Figure 7). It grew the fastest on ammonium nitrate and ammonium chloride with an average growth rate of 0.42 mm day^−1^. The pathogen grew the most slowly on peptone with an average of 0.19 mm day^−1^ (Figure 8a). The colonies sporulated the most heavily on media that contained potassium nitrate followed by ammonium nitrate and ammonium chloride, and the least amount of sporulation was observed on peptone (Figure 8b).

#### 3.3.4. Effects of Temperature on Conidial Germination

**Figure 9:**
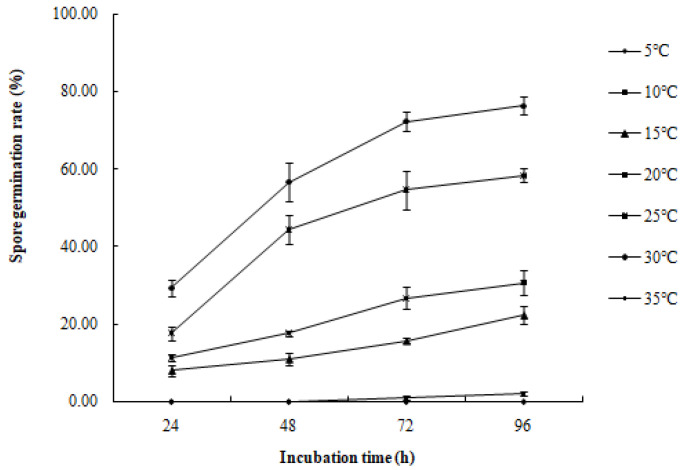
Effect of temperature on the spore germination rate of *R.* *sphaeroidea*.

**Figure 10:**
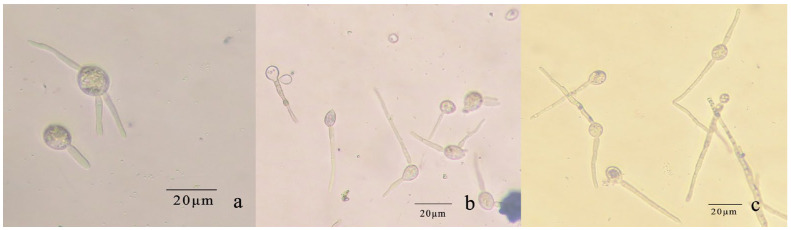
Conidial germination of *R. sphaeroidea.* (**a**) Spores produce 1–3 germ tubes. (**b**,**c**) The elongated bud tube formed hyphae with one or more septa.

The conidia of *R. sphaeroidea* cultured on PDA medium germinated in temperatures from 15 to 30 °C (Figure 9). Spore germination occurred at 24 h at 15–30 °C. The spores produce one to three germ tubes after imbibition (Figure 10a). At 48 h, the bud tube was gradually elongated (Figure 10b). After 72 h, the elongated bud tube formed hyphae with one or more septa (Figure 10c). The optimal temperature for conidial germination was 30 °C. At 96 h after incubation, 30 °C resulted in the highest germination percentage (76.33%). Only a few conidia germinated at 10 °C (2.10%), and no conidia germinated at 5 °C and 35 °C (Figure 9).

### 3.4. Pathogenicity and Host Ranges

**Figure 11:**
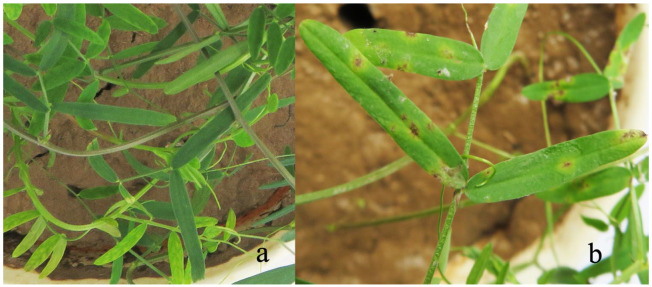
Hairy vetch leaves inoculated with *R. sphaeroidea* after 14 days. (**a**) Control and (**b**) hairy vetch inoculated with conidia.

**Figure 12:**
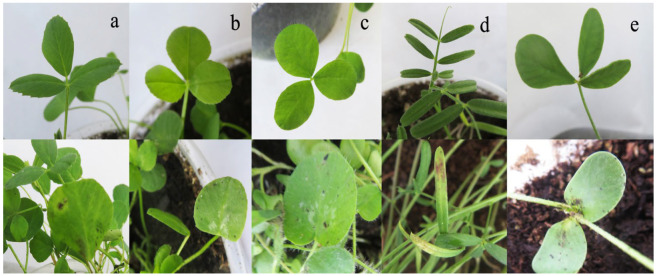
Reaction of five perennial legume plants to *R. sphaeroidea* with irregular brown to dark brown spots 2 weeks after inoculation. (**a**) An alfalfa seedling on the lower panel is inoculated with R. sphaeroidea, and the seedling on the upper panel is the control. (**b**) A white clover seedling with and without inoculation. (**c**) A red clover seedling with and without inoculation. (**d**) A common vetch seedling with and without inoculation. (**e**) A sainfoin seedling with and without inoculation.

After inoculation with YN1931401, brown to dark brown lesions were observed at the edge of the leaves (Figure 11). Moreover, no symptoms appeared on the control plants. *R. sphaeroidea* was re-isolated from the inoculated plants. Two weeks after inoculation with *R. sphaeroidea*, the five perennial legumes, including sainfoin, alfalfa, white clover, red clover, and common vetch, exhibited irregular, brown to dark brown spots on the leaves (Figure 12). Erect milkvetch plants had no apparent symptoms. *R. sphaeroidea* was successfully reisolated from the chlorotic leaves of sainfoin, alfalfa, white clover, red clover, and common vetch plants, and the control plants remained healthy. *R. sphaeroidea* was not isolated from the control.

### 3.5. Fungicide Sensitivity Test

Difenoconazole (10%) was the most effective fungicide with an EC50 value of 319.54 mg L^−1^, and there was a significant difference when compared with the other three fungicides. Furthermore, the EC50 values of chlorothalonil, mancozeb, and difenoconazole were 2743.42, 624.72, and 330.95 mg/L, respectively (Table 2).

## 4. Discussion

This article extends the knowledge of the growth, sporulation, fungicide efficacy, and host range of *R. sphaeroidea*, which had been previously described on *V. fabae* and *V. sativa* in the 19th century [21]. Purple vetch (*V. benhalensis*) and “Lana” woolly pod vetch (*V. villosa* sp. *varia*) in the Salinas Valley (CA, USA) showed a leaf spot disease that was identified as *R. sphaeroidea* [14]. The identification was based largely on morphological characteristics, such as the distinctive shape of the conidia and colonies. The morphological features of *R. sphaeroidea*, such as the conidial shape, also clearly differed from those of the other species of *Ramularia*. The spores of *R. sphaeroidea* are hyaline, spherical, smooth, aseptate, and 2.13 to 3.67 × 4.56 to 5.77 mm (n = 50). The spores of *R. stellenboschensis*, *R. abscondita*, *R. trollii,* and *R. unterseheri* are hyaline, thin-walled, smooth, erect, septate, cylindrical-oblong, and unbranched; in contrast, the spores of *R. sphaeroidea* are spherical [14]. Moreover, the spores of *R. sphaeroidea* were smaller than those described in the USA, possibly owing to climatic differences. The pathogenicity on hairy vetch was also confirmed, and white colonies were often observed on the lower parts of plants at unfavorable temperatures or relative humidity.

The current identification of *Ramularia* is based on the nucleotide sequences of specific gene regions [16]. Phylogenetic analyses conducted based on the combination of seven loci, including ITS, LSU, *cmdA*, *gapdh*, *his3*, *rpb2*, and *tef1-α* sequences and morphological characteristics, clearly distinguished *R. sphaeroidea* (YN1931401, YN1931402, and YN1931403) from the other closely related species of *Ramularia*.

This study describes that *R. sphaeroidea* grew the fastest on OMA, whereas other studies showed that L-malic acid PDA was the optimal media for the mycelial growth of *R. sphaeroidea* isolated from vetches [14]. The most obvious colony characteristic of *R. sphaeroidea* was an irregular bulge with transparent exudates on all media. In the field, after the occurrence of the disease caused by *R. sphaeroidea,* the leaves turned yellow and fell off prematurely. Studies have shown that pathogens such as *Fusarium graminearum* produce the mycotoxin deoxynivalenol (DON), which promotes its expansion during infection of its plant host, wheat [56]. Some compounds can even harm livestock. For example, the pathogen *Alternaria gansuense* causes a systemic disease in a legume and produces swainsonine, an indolizidine alkaloid known as a glycosidase inhibitor. This compound causes an irreversible lysosomal storage disease known as locoism in Western and Northern America, Canada, and China [57]. Further studies should be conducted to determine whether diseased hairy vetch and pure cultures of *R. sphaeroidea* contain toxic substances that lead to this phenomenon.

*Vicia villosa* var. *glabrescens* is a green manure plant that harbors bacteria capable of fixing nitrogen in nodules and spreading over a large area in China [3,9]. Previously, *Vicia villosa* var. *glabrescens*, *V. benhalensis*, *V. villosa* sp. *varia*, *V. fabae*, and *V. sativa* were recognized as hosts for *R. sphaeroidea* [14,23]. This study showed that *R. sphaeroidea* was pathogenic to sainfoin, common vetch, red clover, white clover, and alfalfa when artificially inoculated. These observations indicate that *R. sphaeroidea* can potentially damage other leguminous plants, emphasizing the importance of incorporating it into field management strategies. Further experiments are required to study the infection cycle of *R. sphaeroidea*.

Studies have shown that chemical control is one of the most effective and rapid control methods [58]. Difenoconazole is a triazole fungicide with proven bioefficacy against grapevine powdery mildew disease [59]. In this study, difenoconazole (10%) was found to be the most effective fungicide against leaf diseases caused by *R. sphaeroidea*. The terminal residues of difenoconazole in whole bananas and pulp were 0.45~0.84 mg/kg and 0.19~0.37 mg/kg, respectively, which were lower than the maximum residue level established in China [60].

## 5. Conclusions

In summary, *R. sphaeroidea* grew and produced spores on all the media tested in this study and grew best on OMA, lactose, maltose, ammonium nitrate, and ammonium chloride media. *R. sphaeroidea* can infect other leguminous plants, such as sainfoin, common vetch, red clover, white clover, and alfalfa. The association between *R. sphaeroidea* and a decline in hairy vetch yield and potential control measures to reduce the disease merit further study. Difenoconazole (10%) was the most effective fungicide to control the leaf spot disease caused by *R. sphaeroidea*. It can be widely used to control other *Ramularia* leaf spot diseases.

## Figures and Tables

**Table 1:** Morphological characteristics of different media and carbon and nitrogen sources on *R. sphaeroidea*.

		Colony			Conidia	
		Diameter (mm)	Morphology		Characteristics	Size (μm)
			Upper	Reverse		
Media	PDA	17–20	white	grey to grey-black	hyaline, spherical, smooth, aseptate	2.13–3.67 × 4.56–5.77
	OMA	15–16	white to yellowish	grey	aseptate, ellipsoid to spherical aseptate, spherical	4.27–6.23 × 4.94–6.59
	LA-PDA	13–14	white to pale yellow	white		5.59–6.19 × 5.94–6.41
	PCA	8	pale pink	pale pink	aseptate, spherical to subglobose	4.54–6.78 × 5.51–8.61
	CMA	12	white	pale yellowish with brown margins	aseptate, spherical	4.41–6.22 × 5.06–7.21
Carbon sources	Glucose	10	white yellowish	yellow-brown	orbicular-ovate, spherical,	5.72–6.69 × 4.48–6.89
	Maltose	8.5	yellowish with no aerial mycelium	yellowish	hyaline, thin-walled, smooth, oval	5.82–8.50 × 4.53–6.59
	Lactose	15	yellow with a white thin edge, black raised	pale yellow with black raised	subglobose	3.84–6.36 × 3.58–6.60
	Starch	6	yellow with black in the center	pale brown	orbicular-ovate	5.03–6.74 × 4.96–5.87
	D-fructose	13	white with yellow, thin	black at the center and pale yellowish	spherical	7.47–8.58 × 8.6–7.97
	Sucrose	12	black at the center with yellowish edges	yellow	spherical	3.25–5.22 × 3.63–5.13
Nitrogen sources	Potassium nitrate	7	white aerial mycelium, pale pink with a yellow-brown edge on the upper side	Brown-black	spherical	4.23–7.54 × 5.22–7.24
	Ammonium nitrate	9.5	pale pink with white aerial mycelium, yellow-brown with black edge	pale brown in the center with a black halo	ellipsoidal to obovoid	5.45–7.78 × 5.08–7.49
	Peptone	6.5	white with pale-brown edge	white	obovoid, spherical	4.86–7.65 × 5.86–7.16
	Ammonium chloride	11	pink with a pale-yellow edge, no aerial mycelium	pale brown in the center with a pale pink halo	spherical	4.23–7.64 × 4.39–7.74

**Table 2:** Inhibitory effects of different fungicides on *R. sphaeroidea*.

Fungicides	Regression Equation	Correlation Coefficient	EC50(mg/L)
Chlorothalonil 75%	y = 0.0201x + 0.5688	0.8131	2743.42
Mancozeb 80%	y = 0.017x + 0.748	0.9434	624.72
Difenoconazole 10%	y = 0.006x + 1.4135	0.8696	319.54
Pentazole alcohol 50%	y = 0.0061x + 1.4124	0.9951	330.95

## Data Availability

The data that support the findings of this study are available in GenBank (https://www.ncbi.nlm.nih.gov/ accessed on 9 March 2022).

## Supplementary Materials

The following supporting information can be downloaded at: https://www.mdpi.com/article/10.3390/microorganisms12040766/s1, Table S1: Collection details and GenBank accession numbers in this study. References [49,50,51,52,53] are cited in the Supplementary Materials.
