# Supplementary material for: Evaluation of the Growth, Sporulation, Fungicide Efficacy, and Host Range of Ramularia sphaeroidea
Source: Microorganisms. 2024 Apr 10;12(4):766. doi: 10.3390/microorganisms12040766 (PMC11051774; doi:10.3390/microorganisms12040766)
Supplement: Supplementary file 1 [file microorganisms-12-00766-s001.zip › microorganisms-2923922-supplementary.pdf]

microorganisms

MDPI

Table S1. Collection details and GenBank accession numbers in this study.

Species	Culture numbers [a]	Host	Country	GenBank accessions[b] ITS	LSU	*CmdA*	*rpb2*	*gapdh*	*tef1-α*	*his3*	References
Ramularia abscondita	CBS 114727	*Arctium*	Sweden	KX287307	KX287006	KX289026	KX288465	KX288143	KX287864	KX288753	[16]
R. acroptili	CBS 120252	*Acroptilon*	Turkey	GU214689	GU214689	KX289027	KX288472	KX288150	KX287871	KX288760	[16]
R. agastaches	CPC 10820	*Agastache*	South Korea	KX287317	KX287016	KX289029	KX288476	KX288154	KX287875	KX288764	[16]
	CPC 10821	*Agastacherugosa*	South Korea	KX287318	KX287017	KX289030	KX288477	KX288155	KX287876	KX288765	[16]
R. archangelicae	CBS 108992	*Angelica sylvestris*	Austria	KX287329	KX287028	KX289035	KX288488	KX288166	KX287887	KX288776	[16]
	CBS 109011	*Angelica sylvestris*	Austria	KX287330	KX287029	KX289036	KX288489	KX288167	KX287888	KX288777	[16]
R. asteris	CBS 131.21	*Aster tripolium*	Netherlands	KX287334	KX287034	KX289039	KX288494	KX288172	KX287893	KX288782	[16]
R. beticola	CPC 30065	*Beta vulgaris*	Denmark	KX287337	KX287037	KX289040	KX288496	KX288175	KX287896	KX288785	[16]
	CPC 30066	*Beta vulgaris*	France	KX287338	KX287038	KX289041	KX288497	KX288176	KX287897	KX288786	[16]
R. buniadis	CBS 114301	*Bunias orientalis*	Sweden	KX287347	KX287047	KX289048	KX288506	KX288185	KX287906	KX288795	[16]
R. calcea	CBS 101612	*Symphytum* sp.	Germany	KP894219	KP894111	KP894879	KP894657	KP894546	KP894436	KP894768	[51]
	CBS 114442	*Viola hirta*	Sweden	KP894229	KP894122	KP894884	KP894668	KP894557	KP894447	KP894779	[49]
R. carneola	CBS 108975	*Scrophularia nodosa*	Netherlands	KX287348	KX287048	KX289049	KX288507	KX288186	KX287907	KX288796	[16]
	CBS 108976	*Scrophularia nodosa*	Netherlands	KX287349	KX287049	KX289050	KX288508	KX288187	KX287908	KX288797	[16]
R. chamaedryos	CBS 116577	*Veronica chamaedrys*	Sweden	KX287353	KX287053	KX289054	KX288512	KX288192	KX287912	KX288802	[16]
	CBS 118794	*Veronica persica*	New Zealand	KX287356	KX287056	KX289055	KX288515	KX288195	KX287915	KX288805	[16]
R. chelidonii	CPC 12208	*Hylomecon vernalis*	South Korea	KX287358	KX287058	KX289056	KX288517	KX288197	KX287917	KX288807	[16]
R. collo-cygni	CBS 101181	*Hordeum vulgare*	Germany	KJ504786	KJ504745	KJ504513	KJ504657	KJ504569	KJ504701	KJ504613	[42]
	CBS 101182	*Hordeum vulgare*	Germany	KX287386	KX287086	KX289058	KX288544	KX288224	KX287945	KX288834	[16]
R. coryli	CBS 117800	*Corylus avellana*	Netherlands	KX287391	KX287091	KX289061	KX288549	KX288229	KX287950	KX288839	[16]
R. deusta	CBS 473.50	*Lathyrus latifolius*	Guadeloupe	KX287399	KX287101	KX289062	KX288559	KX288239	KX287960	KX288849	[16]
R. digitalis-ambiguae	CBS 434.67	*Digitalis purpurea*	Luxembourg	KX287407	KX287109	KX289063	KX288566	KX288247	KX287968	KX288856	[16]
R. endophylla	CBS 113871	*Quercus robur*	Netherlands	KP894237	KP894130	KP894891	KP894677	KP894566	KP894455	KP894787	[49]
	CBS 113265	*Quercus robur*	Netherlands	AY490763	AY490776	KF253981	KP894673	KP894562	KF253276	KP207603	[50]
	CBS 101680	*Castanea sativa*	Netherlands	KP894233	KP894126	KP894887	KP894672	KP894561	KP894451	KP894783	[49]
R. eucalypti	CBS 120728;	*Eucalyptus* sp.	Australia,	KJ504793	KJ504751	KJ504520	KJ504664	KJ504576	KJ504708	KJ504620	[42]
R. gei	CBS 344.49	*Geum urbanum*	Netherlands	KX287411	KX287113	KX289064	KX288570	KX288251	KX287972	KX288860	[16]
	CBS 113977	*Geum* sp.	Sweden	KX287412	KX287114	KX289065	KX288571	KX288252	KX287973	KX288861	[16]
R. geranii	CBS 159.24	*Geranium pyrenaicum*	France	KX287413	KX287115	KX289066	KX288572	KX288253	KX287974	KX288862	[16]
		Geranium sylvaticum	France	KX287414	KX287116	KX289067	KX288573	KX288254	KX287975	KX288863	[16]
R. glennii	CBS 129441T	*Human bronchia*	Netherlands	KJ504769	KJ504728	KJ504500	KJ504640	KJ504552	KJ504684	KJ504596	[42]
	CBS 122989	Human skin	Netherlands	KJ504768	KJ504727	KJ504499	KJ504639	KJ504551	KJ504683	KJ504595	[42]
R. grevilleana	CBS 719.84	*Fragaria × ananassa Tioga*	New Zealand	EU167605	KP894116	KP894881	KP894662	KP894551	KP894441	KP894773	[51]
	CBS 298.34	-	Netherlands	KP894223	KP894115	KP894880	KP894661	KP894550	KP894440	KP894772	[49]
R. heraclei	CPC 11505	*Heracleum moellendorffii*	South Korea	KX287423	KX287125	KX289071	KX288582	KX288263	KX287984	KX288870	[16]
	CPC 11506	*Heracleum moellendorffii*	South Korea	KX287424	KX287126	KX289072	KX288583	KX288264	KX287985	KX288871	[16]
	CPC 11507	*Heracleum moellendorffii*	South Korea	KX287425	KX287127	KX289073	KX288584	KX288265	KX287986	KX288872	[16]
R. hydrangeae-macrophyllae	CBS 122273	*Hydrangea macrophylla*	New Zealand	KX287433	KX287135	KX289077	KX288592	KX288273	KX287994	KX288880	[16]
	CBS 118410	*Ligularia clivorum*	New Zealand	KX287435	KX287137	KX289078	KX288594	KX288275	KX287996	KX288882	[16]
	CBS 122625	*Iris*	New Zealand	KX287437	KX287139	KX289079	KX288596	KX288277	KX287998	KX288884	[16]
R. inaequalis	CPC 15815	*Taraxacum* sp.	Mexico	KX287457	KX287159	KX289092	KX288616	KX288297	KX288018	KX288904	[16]
	CBS 250.96	*Taraxacum officinale*	Canada	KP894224	KP894117	KP894882	KP894663	KP894552	KP894442	KP894774	[49]
	CPC 15753	*Taraxacum officinale*	Canada	KP894226	KP894119	KP894883	KP894665	KP894554	KP894444	KP894776	[49]

R. leonuri	CPC 11312	*Leonurus sibiricus*	South Korea	KF251331	KF251835	KF253983	KX348080	KX288303	KF253178	KX288910	[52]
	CPC 11314	*Leonurus sibiricus*	South Korea	KX287465	KX287167	KX289095	KX288623	KX288305	KX288025	KX288912	[16]
	CPC 11411	*Leonurus sibiricus*	South Korea	KX287466	KX287168	KX289096	KX288624	KX288306	KX288026	KX288913	[16]
R. lethalis	CBS 141113;	*Acer pseudoplatanus*	Netherlands	KX287472	KX287174	KX289097	KX288630	KX288312	KX288032	KX288919	[16]
R. ligustrina	CBS 379.52	*Ligustrum vulgare*	Italy	KX287473	KX287175	KX289098	KX288631	KX288313	KX288033	KX288920	[16]
R. mali	CBS 129581T	Apple in cold storage	Italy	KJ504778	KJ504737	KJ504506	KJ504649	KJ504561	KJ504693	KJ504605	[42]
R. malicola	CBS 119227	*Malus* sp.	USA	AY598873	AY598910	KX289099	KX288635	KX288316	KX288036	KX288924	[53]
R. miae	CBS 120121	*Wachendorfia thyrsifolia*	South Africa	KJ504801	DQ885902	KJ504525	KJ504672	KJ504584	KJ504716	KJ504628	[42]
	CPC 19770	*Leonotis leonurus*	South Africa	KJ504805	KJ504762	KJ504528	KJ504676	KJ504588	KJ504720	KJ504632	[42]
	CPC 19835	*Gazania rigens* var.*uniflora*	South Africa	KJ504804	KJ504761	KJ504527	KJ504675	KJ504587	KJ504719	KJ504631	[42]
R. neodeusta	CPC 13568	*Lathyrus odoratus*	New Zealand	KX287478	KX287180	KX289100	KX288637	KX288318	KX288038	KX288926	[16]
	CBS 141115	*Vicia faba*	New Zealand	KX287479	KX287181	KX289101	KX288638	KX288319	KX288039	KX288927	[16]
R. helminthiae	CPC 11502	*Picris hieracioides* var. *glabrensis*	South Korea	KX287480	KX287182	KX289102	KX288639	KX288320	KX288040	KX288928	[16]
	CPC 11504	*Picris hieracioides var. glabrensis*	South Korea	KX287481	KX287183	KX289103	KX288640	KX288321	KX288041	KX288929	[16]
R. nyssicola	CBS 127665	*Nyssa* ogeche	USA	KJ504765	KJ504724	KJ504496	KJ504636	KJ504548	KJ504680	KJ504592	[42]
	CBS 127664	*Nyssa ogeche*	USA	KP894231	KP894124	KP894885	KP894670	KP894559	KP894449	KP894781	[49]
R. osterici	CBS 141116	*Ostericum koreanum*	South Korea	KX287483	KX287185	KX289105	KX288642	KX288323	KX288043	KX288931	[16]
	CPC 10751	*Ostericum koreanum*	South Korea	KX287484	KX287186	KX289106	KX288643	KX288324	KX288044	KX288932	[16]
	CPC 10752	*Ostericum koreanum*	South Korea	KX287485	KX287187	KX289107	KX288644	KX288325	KX288045	KX288933	[16]
R. parietariae	CBS 123730;	*Parietaria officinalis*	Czech Republic	KX287486	KX287188	KX289108	KX288645	KX288326	KX288046	KX288934	[16]
	CBS 123731	*Parietaria officinalis*	Czech Republic	KX287487	KX287189	KX289109	KX288646	KX288327	KX288047	KX288935	[16]
R. phacae-frigidae	CBS 234.55	*Phaca frigida*	Switzerland	KP894232	KP894125	KP894886	KP894671	KP894560	KP894450	KP894782	[49]
R. plurivora	CBS 118743T	Human bone marrow	Netherlands	KJ504780	KJ504739	KJ504508	KJ504651	KJ504563	KJ504695	KJ504607	[42]
	CPC 16123	Melon in storage	Netherlands	KJ504782	KJ504741	KJ504510	KJ504653	KJ504565	KJ504697	KJ504609	[42]
R. pratensis var. pratensis	CBS 122105	*Rumex* sp.	Taiwan	KX287488	KX287190	KX289110	KX288647	KX288328	KX288048	KX288936	[16]
R. rubella	CPC 15748	*Rumex obtusifolius*	New Zealand	KX287496	KX287198	KX289111	KX288656	KX288336	KX288056	KX288945	[16]
	CBS 114440	*Rumex longifolius*	Sweden	KX287497	KX287199	KX289112	KX288657	KX288337	KX288057	KX288946	[16]
R. rumicicola	CBS 141118	*Rumex crispus*	South Korea	KX287503	KX287205	KX289115	KX288663	KX288344	KX288064	KX288953	[16]
R. rumicis	CBS 114300	*Rumex aquaticus*	Sweden	KJ504787	KJ504746	KJ504514	KJ504658	KJ504570	KJ504702	KJ504614	[42]
R. vizellae	CBS 115981	*Malus* dead leaf litter	Netherlands	KP894283	KP894176	KP894928	KP894722	KP894611	KP894501	KP894833	[49]
	CBS 115982	*Malus* dead leaf litter	Netherlands	KP894284	KP894177	KP894929	KP894723	KP894612	KP894502	KP894834	[49]
	CBS 117871	*Quercus rubra*	Netherlands	KP894295	KP894188	KP894939	KP894734	KP894623	KP894513	KP894845	[49]
R. stellariicola	CBS 130592	*Stellaria aquatica*	South Korea	GU214693	KX287216	KX289117	KX288673	KX288355	KX288076	KX288965	[44]
R. tovarae	CBS 113305	*Polygonum filiforme*	South Korea	KJ504807	KJ504764	KJ504529	KJ504678	KJ504590	KJ504722	KJ504634	[42]
R. tricherae	CBS 108973	*Knautia arvensis*	Netherlands	KP894249	KP894142	KP894902	KP894688	KP894577	KP894467	KP894799	[49]
R. vizellae	CBS 117871	*Quercus rubra*	Netherlands	KP894295	KP894188	KP894939	KP894734	KP894623	KP894513	KP894845	[49]
R. unterseheri	CBS 124846	*Fagus sylvatica*	Germany	KP894267	KP894160	KP894920	KP894706	KP894595	KP894485	KP894817	[49]
R. sphaeroidea	**YN1931401**	***Vicia villosa***	**China**	**MW092181**	**OM945870**	**OM981227**	**OM981230**	**MW448178**	**MW448172**	**MW448175**	**This study**
R. sphaeroidea	**YN1931402**	***Vicia villosa***	**China**	**MW332205**	**OM945871**	**OM981228**	**OM981231**	**MW448179**	**MW448173**	**MW448177**	**This study**
R. sphaeroidea	**YN1931403**	***Vicia villosa***	**China**	**MW332206**	**OM945872**	**OM981229**	**OM981232**	**MW448180**	**MW448174**	**MW448177**	**This study**
R. sphaeroidea	CBS 112891	*Vicia villosa*	USA	AY352584	KX287215	KX289117	KX288673	KX288354	KX288074	KX288963	[14]
R. vallisumbrosae	CBS 271.38	*Narcissus Victoria*	UK	KX287239	KX287224	KX289121	KX288698	KX288378	KX288098	KX288988	[16]

R. urticae	CBS 105.26	*Urtica dioica*	Sweden	KP894276	KP894168	KP894926	KP894714	KP894604	KP894494	KP894826	[49]
Mycosphaerelloides madeirae	CBS 116066	*Quercus robur*	Netherlands	AY853188	KX286989	KX289024	KX288444	KX288139	KX287855	KX288747	[49]

a Culture numbers in bold represent ex-type specimens. MHLZU, Mycological Herbarium of Lanzhou University. b ITS = internal transcribed spacer 1 and 4 together with 5.8S nrRNA; LSU = partial 28S nrRNA gene; CmdA = calmodulin gene; rpb2 = RNA polymerase II second largest subunit; gapdh = glyceraldehyde-3-phosphate dehydrogenase gene; tef1-α = translation elongation factor 1-α gene; and his3 = histone H3 gene.

Reference

14. Koike, S.T.; Smith, R.F.; Crous, P.W.; Groenewald, J.Z. Leaf and stem spot caused by *Ramularia sphaeroidea* on purple and lana woollypod vetch (*Vicia* spp.) cover crops in California. *Plant Dis.* **2004**, *88*, 221–221. https://doi.org/10.1094/Pdis.2004.88.2.221b.

16. Videira, S.; Groenewald, J.Z.; Braun, U.; Shin, H.D.; Crous, P.W. All that glitters is not *Ramularia*. *Study Mycol.* **2016**, *83*, 49–163. https://doi.org/10.1016/j.simyco.2016.06.001.

42. Videira, S.; Groenewald, J.Z.; Kolecka, A.; Haren, L.V.; Boekhout, T.; Crous, P.W. Elucidating the *Ramularia eucalypti* species complex. *Persoonia* **2015**, *34*, 50–64. https://doi.org/10.3767/003158515X685670.

49. Videira, S.I.R.; Groenewald, J.Z.; Verkley, G.J.M.; Braun, U.; Crous, P.W. The rise of *Ramularia* from the *Mycosphaerella labyrinth*. *Fungal Biol.* **2015**, *119*, 823–843.
https://doi.org/10.1016/j.funbio.2015.06.003.

50. Verkley, G.J.; Crous, P.W.; Groenewald, J.E.; Braun, U.; Aptroot, A. *Mycosphaerella punctiformis* revisited: Morphology, phylogeny, and epitypification of the type species of the genus *Mycosphaerella* (Dothideales, Ascomycota). *Mycol. Res.* **2004**, *108*, 1271–1282. https://doi.org/10.1017/S0953756204001054.

51. Simon, U.K.; Groenewald, J.Z.; Crous, P.W. *Cymadothea trifolii*, an obligate biotrophic leaf parasite of *Trifolium*, belongs to Mycosphaerellaceae as shown by nuclear ribosomal DNA analyses. *Persoonia-Mol. Phylogeny Evol. Fungi* **2009**, *22*, 49–55. https://doi.org/10.3767/003158509X425350.

52. Verkley, G.J.M.; Quaedvlieg, W.; Shin, H.D.; Crous, P.W. A new approach to species delimitation in *Septoria*. *Stud. Mycol.* **2013**, *75*, 213–305. https://doi.org/10.3114/sim0018.

53. Esra, G.Ü.L.; Karatas, Z.; Karakaya, A. Evaluation of the fungicide resistance of gray mold (*Botrytis cinerea*) in tomatoes to boscalid and pyraclostrobin in greenhouse areas of Turkey. *J. Agric. Sci.* **2021**, *31*, 487–493. https://doi.org/10.29133/yyutbd.820029.
